# Application of network link prediction in drug discovery

**DOI:** 10.1186/s12859-021-04082-y

**Published:** 2021-04-12

**Authors:** Khushnood Abbas, Alireza Abbasi, Shi Dong, Ling Niu, Laihang Yu, Bolun Chen, Shi-Min Cai, Qambar Hasan

**Affiliations:** 1 School of Computer Science and Technology, Zhoukou Normal University, Zhoukou, 466001 China; 2 School of Engineering and Information Technology, University of New South Wales, Canberra, NSW 2006 Australia; 3 School of Computer Science and Engineering, University of Electronic Science and Technology of China, Chengdu, 610054 China; 4 College of Computer and Software Engineering, Huaiyin Institute of Technology, Huaian, 223003 China; 5 Centre for Cellular and Molecular Biology, School of Life and Environmental Science, Deakin University, Burwood, VIC 3125 Australia

**Keywords:** Data-driven drug discovery, Network link prediction, Poly-pharmacy, Poly-pharmacy side effects prediction, Drug-target prediction

## Abstract

**Background:**

Technological and research advances have produced large volumes of biomedical data. When represented as a network (graph), these data become useful for modeling entities and interactions in biological and similar complex systems. In the field of network biology and network medicine, there is a particular interest in predicting results from drug–drug, drug–disease, and protein–protein interactions to advance the speed of drug discovery. Existing data and modern computational methods allow to identify potentially beneficial and harmful interactions, and therefore, narrow drug trials ahead of actual clinical trials. Such automated data-driven investigation relies on machine learning techniques. However, traditional machine learning approaches require extensive preprocessing of the data that makes them impractical for large datasets. This study presents wide range of machine learning methods for predicting outcomes from biomedical interactions and evaluates the performance of the traditional methods with more recent network-based approaches.

**Results:**

We applied a wide range of 32 different network-based machine learning models to five commonly available biomedical datasets, and evaluated their performance based on three important evaluations metrics namely AUROC, AUPR, and F1-score. We achieved this by converting link prediction problem as binary classification problem. In order to achieve this we have considered the existing links as positive example and randomly sampled negative examples from non-existant set. After experimental evaluation we found that *Prone*, *ACT* and $LRW_5$ are the top 3 best performers on all five datasets.

**Conclusions:**

This work presents a comparative evaluation of network-based machine learning algorithms for predicting network links, with applications in the prediction of drug-target and drug–drug interactions, and applied well known network-based machine learning methods. Our work is helpful in guiding researchers in the appropriate selection of machine learning methods for pharmaceutical tasks.

**Supplementary Information:**

The online version supplementary material available at 10.1186/s12859-021-04082-y.

## Background

Diseases are a complex set of phenomena that include non-linear relationships between an individual cell and an organism [1]. To find the proper response against a particular disease, i.e. designing and developing appropriate drugs, requires consideration of many such phenomena [2]. The traditional drug discovery processe is expensive involving five steps [3–8]: (1) discovery and pre-clinical research; (2) safety review; (3) clinical research; (4) regulatory review (e.g., by the American Food and Drug Administration, FDA); and (5) regulatory post-market safety monitoring. That makes it time-consuming [5] and costly [9]. However, data-driven computerised drug discovery methods offers the potential to speed up the drug discovery process. There are currently four categories of methods for data-driven drug discovery: (1) ligand-based approaches; (2) docking approaches; (3) network-based approaches; and (4) machine learning-based approaches. Ligand-based target prediction assumes that similar drugs tend to bind similar targets (e.g. diseases). As this approach utilises the similarity of the ligands for making prediction, it requires examples of interactions between drugs and targets for prediction. Docking-based methods make predictions based on the three-dimensional structure of proteins, and is greatly limited when the structure of the protein is unknown. However, network-based and machine learning-based approaches attempt to overcome the aforementioned limitations of the other two approaches [10]. Nevertheless, not only ligand- and docking-based prediction approaches require prior data, but also network- and machine-learning approaches depend on reliable prior data (training data for ML, relevant interaction data for network approaches).

Here, we formulate a data-driven drug discovery approach that models drug-target interactions (DTI) as networks between two sets of nodes: the drug candidates, and the entities affected by the drugs (i.e. diseases, genes, and other drugs), which are referred to as targets. Our aim is to predict the missing nodes (i.e. drugs and targets) and links between them. For example, we attempt to predict which candidate drugs might treat a list of diseases. There is a large amount of data identifying which drugs treats which diseases, but some diseases have very few drugs available. Thus, discovering which existing drugs can treat them is of great importance. Further, it is critical to determine which drugs have side effects in the presence of other drugs as interactions among drugs may be harmful or lethal to patients. Therefore, drug interaction networks are considered to predict what is the likelihood of reactions between combinations of drugs in a patient’s body. Likewise, we formulate a drug-target interaction network to predict missing links between drugs and target diseases.

When considering *n* drugs, then there will be $n*(n-1)/2$ combinations of drug–drug relationships for trials. Because a patient could be taking more than two medicines together, the resulting combinations are of an even higher-order and are not feasible to test via experiments. Thus, link prediction offers an important solution. Besides, it allows to find additional uses of existing drugs, with 30% of 84 drugs introduced in 2013 being reused. Many drugs affect more than one particular protein or gene, and some medical conditions involve multiple genes and proteins. Modeling such situations as network interactions and formulating a link prediction problem enables drug-target gene prediction.

Traditional machine learning approaches applied to the drug–target interaction (DTI) problem have many constraints, including dimensionality (for complex and large pharmacological datasets) and incompleteness [11], sparsity, and heterogeneity (mainly in biological datasets). For instance, logistic regression and support vector machines suffer from the high dimensionality and numerous implicit relationships in the data. These are the result of many factors including measurement technologies [12] and bias problems during the recording of the data [13]. Besides, the spreading speed of diseases or other causes of infection such as viruses evolve quickly is not considered in traditional machine learning methods. In addition, the hierarchical nature of biological data (connections among genes, proteins, and so on) cannot be easily modeled by traditional machine learning approaches. Therefore, there is a need for methods and models capable of addressing these problems.

**Fig. 1:**
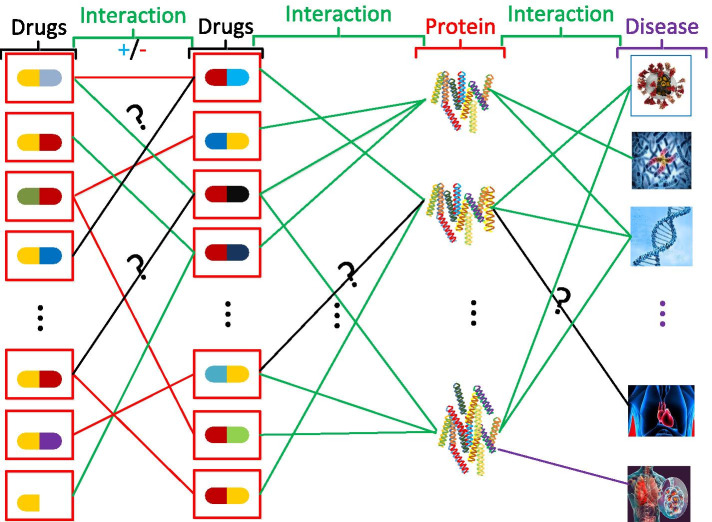
A depiction of the link prediction approach in drug discovery. This is a heterogeneous drug-target and drug–drug interaction network

Network-based approaches are gaining attention because of their simplicity (node and edge representation) which effectively considers high dimensionality and heterogeneity as well as implicit relationships. For drug discovery, these relationships include sharing a common chemical formula and structure or affecting the same protein. Such ability supports reusing existing drugs in new ways, as with a recent breast cancer treatment [14]. As noted previously, this accelerates the drug discovery process, saving time and expense [15]. As an example, other medications such as Duloxetine, used for treating depression, have been found powerful in treating urine leakage issues [16]. Thus, we can consider drug discovery as a missing link problem between chemicals and proteins as shown in Fig. 1.

This work exploits network-based link prediction models for solving the following pharmaceutical problems:

- Drug–target interaction prediction: This task is to predict which drug will affect which protein this is one of the application in drug repurposing.
- Drug–drug side effect prediction: From existing drug–drug side effect data, we can create a network, in which a link reflects the two drugs (nodes) has shown some side effects. So in this task we predict which new drug combinations can cause side effect.
- Disease–gene association prediction: Some disease affects the genes which is more lethal as it can transfer to the next generations. Therefore in this task we aim to predict which new disease can affect which particular gene.
- Disease–drug association prediction: Some drugs might not be pharmaceutical chemicals such as arsenic. So in this problem, we aim to predict which drug is associated to which disease.

We also evaluate the performance of the models using five publicly available pharmacological datasets, and report the performance of these models according to three different evaluation metrics.

### Network-based approaches for drug discovery

Researchers have also explored network topology-based link prediction methods for drug–target interaction (DTI) prediction. Pech et al. [17] propose a sparse learning method for link prediction. Fokoue et al. [18] propose a knowledge graph DTI prediction framework called *Tiresias*. Chen et al. [19] presented Network-based Random Walk with Restart on the Heterogeneous network (NRWRH), based on random walk with restart on a heterogeneous network, by constructing the drug similarity, protein similarity, and drug–target network as a heterogenous network. Cheng et al. and Huang et al. [20, 21] solve the DTI problem using a bipartite network and proposed three DTI prediction methods: drug-based similarity inference (DBSI), target-based similarity inference (TBSI), and network-based inference (NBI). The drug side effect similarity inference (DSESI) method [22] utilises the drug–drug chemical similarity and the phenotypic side effect similarity. The multiple target optimal intervention (MTOI) method [23] solves this problem with two steps: finding the known drug–target links, and applying a multiple target control inference mechanism. Luo et al. [24] combine various attributes from heterogeneous networks and propose a novel network integration pipeline, DTINet for DTI prediction. This solution utilises drug and protein distributions in each network and embeds the high dimensional protein and drug data into lower dimensions. Another proposal suggests a meta-path-based methodology to separate the semantic highlights of DTIs from heterogeneous networks [25].

Figure 1 shows how a drug discovery problem can be converted to a link prediction problem. The relationship network is heterogeneous as many entities are related, such as drug–drug, drug–gene, drug–disease, disease–gene, and drug–drug side effects (see Fig. 1). However, we consider only monopartite and bipartite individual networks in our study.

### Deep learning based approaches for drug discovery

Duvenaud et al. [26] present a deep learning model for generating molecular features based on convolutional neural networks. Gilmer et al. [27] propose a deep learning framework using a message passing neural network for molecular property prediction. You et al. [28] propose a Reinforcement Learning-based Graph Convolutional Policy Network (GCPN) as a goal-directed graph generation model. This approach is highly applicable to both chemistry and drug discovery where the goal is to find new molecules with given molecular properties such as drug similarity and synthetic accessibility. Cao et al. [29] propose a Generative Adversarial Network (GAN) generative approach that supports creating molecules with desired molecular properties. Coley et al. [30] and Kearnes et al. [31] solve molecular graph representation problems by applying a graph convolutional network to an undirected molecular graph. Along with molecular graph structural attributes, they also consider other factors such as atom and bond attributes, neighbouring atoms, and radii. Xie et al. [32] propose a Crystal Graph Convolutional Neural Network framework that is able to learn material properties from the crystal atomic link structure, which can be very helpful in new material design. Ktena et al. [33] use graph convolutional neural networks for graph similarity prediction to identity brain disorders. Parisot et al. [34, 35] use a graph convolutional network for brain disease prediction. Assouel et al. [36] propose a conditional graph generative model.

### Genomic and phenotypic study for drug discovery

Advances in the study of genomes have generated huge volumes of genomic and transcriptomic data, including a diverse set of disease samples, standard tissue samples, and cell lines. Gene expression data from these studies have been widely adopted for research purposes. One of the widely adopted genomic datasets is the Library of Integrated Network-based Cellular Signatures (LINCS) [37] that contains extensive data from cancer cell lines treated under different conditions. One benefit of using genomic data is ’signature reversion’ that enables the study of reverse relationships as well, i.e., drug–disease and disease–drug. The other area of study of computational drug discovery is phenotypic information, in which a study of the phenome is performed to identify the genetic association with disease [38]. This is also known as a phenome-wide association study. For example, Bisgin et al. [39] use phenotypic information from the Side Effect Resource (SIDER) database [40] and applied Dirichlet Allocation Model for drug re-positioning discovery. Phenotype information can be used to make other kinds of association predictions also. For example, some researchers used phenotypic link prediction between drug–gene and phenotype–disease information [41].

### Role of drug chemical structure in drug discovery

Another computational method for drug discovery is examination of the chemical structure of drugs by representing the molecular structure as a network. Hence, this approach is based on the assumption that compounds with similar structures will act similarly against the same proteins. Other methods make use of the molecular structures themselves, as with 2D topological fingerprints and 3D informatics. Researchers such as Swamidass et al. [42] study which chemical structures modulate which disease relevant phenomes. This helps predict what other drugs will affect the same protein or disease. Tan et al. [43] employ chemical structures along with semantic gene similarity to construct a drug similarity network, which can be then used to find novel drug–target relations. Further, Ng. et al. [44] propose ligand Enrichment of Network Topological Similarity (ligENTS) to identify novel drug–target relations by using the chemical structure of the drug for drug repurposing task.

### Study of drug combinations in drug discovery

Another area of work is drug combination prediction as many diseases are the results of complex events involving many complex molecular structures. Predicting interactions between multiple molecules and processes is thus important. In many cases, more than one drug is used to treat diseases, such as diabetes, cancer, and bacterial infections, as the drug combinations are found to be more effective than single drug therapies [45, 46]. For example, B-cell lymphoma (DLBCL) is a malignant cancer requiring multiple targeted drugs, some or all of which are administered at one time. However, the presence of one drug sometimes increases or decreases the effects of other drugs. Combinations can even cause fatalities from Adverse Drug Reactions (ADRs), which are the fourth leading cause of death in the United States [47]. Such interactions are usually not observed during clinical trials of individual drugs as testing of combinations in vivo is both time consuming and expensive.

Therefore, the problem of drug combinations can be formulated as two types of the link prediction problem, namely treating disease, and side effects and reactions. In Figure 1 shows these links with red and green colors, respectively. Drug–drug side effect prediction is an important task in its own right, but some researchers like Campillos et al. [22] use drug side effects as features for predicting novel target prediction. Further, Zitnik et al. [48] propose Dacagon, a graph convolutional network-based framework, to predict which drug combinations cause which side effects in patients. Li et al. [49] develop a bipartite drug–target network to find similar drugs using a graph node similarity approach. Li et al. [50] develop a multilayered network of gene–disease and drug–target network to identify new therapeutic uses of existing drugs. Wu et al. [51] formulate a drug–disease heterogeneous network to identify similar drug and disease pairs. Jin et al. [52] develop a novel method to identify similar drugs for cancer by exploiting off-target effects which act on important cancer cell signalling pathways. This approach employs a model called the Bayesian Factor Regression Model (BFRM), introduces a new network component called Cancer Signalling Bridges (CSB), and integrates the two into a hybrid method called CSB-BFRM. The researchers have applied this approach to breast and prostate cancer cells.

## Methodology and data

### Problem description: the drug discovery problem

We investigate the drug discovery problem by applying various contemporary models to different pharmacological situations to see how well the prediction models are able to facilitate drug discovery process. To carry out our task, we first build the networks, either mono-partite or bipartite, and ensured they were undirected. We convert bipartite datasets into the corresponding biadjacency matrix, and then apply the link prediction models to solve the following drug discovery problems:

1. Drug–Target prediction: The aim is to predict which drug will affect which unknown proteins, considering the bipartite networks of drugs and their target proteins.
2. Drug–Disease prediction: The aim is to study drug chemical structures and target proteins (as the disease and drugs both affects proteins) to find similarities between drug structures. It has been found that similar drug structures affect similar proteins. We represent each chemical structure as a network. Once a similar drug is found, it can be used to target similar proteins. There is currently a lack of systematic research in this area.
3. Drug–Drug reaction prediction: This problem examines the search for combinations of drugs for conditions that require targeting more than one protein, as with degenerative neurological conditions, such as Alzeheimer’s and Parkinson’s. We incorporate known combinations that cause adverse side effects (headaches, vomiting, rashes, etc.) to predict which additional combinations might cause reactions in patients.
4. Disease–Gene association prediction: Thanks to high-throughput screening technologies, we have large volumes of genomic data. Yet, there are many diseases for which a genomic basis is unknown. Genomic alleles and malignant mutations are continuously sequenced, which is why most of them are identified or annotated. Traditionally, linkage analysis has been done to find non-experimental disease-gene associations, and it has been based on the likelihood of observing alleles. However, this kind of analysis fails for a multifactorial and heterogeneous diseases. Considering genomic data association is a newer approach to solve this problem, but with the downside of producing hundreds of candidates for complex diseases, which hinders experimental validation. Therefore, we present a network approach for genomic data analysis. In addition to existing network approaches, such as common neighbour, path-based and random walk-based methods, recent developments in network-based learning technologies, such as geometric deep learning, offer the prospect of using genomic data to find gene-disease associations that are still unknown.

### Link prediction models for drug discovery

Given a set of nodes *V* and a set of edges *E*, the corresponding network is *G*(*V*, *E*) at time *t*. The graph *G* can be effectively represented by a ($\mathrm{{|v|}} \times \mathrm{{|v|}}$) adjacency matrix *A* where the entry $A_{ij}=w$ is non-zero if there is a link between node *i* and *j*, and 0 otherwise. Many problems in drug discovery can be modelled as mono-partite undirected networks (e.g., drug–drug networks), but some such as drug–target networks require bi-partite models, i.e. having two different sets of nodes: drugs and targets. In this case, the link relationship can be represented as a $B_{r \times k}$ biadjacency network [53] whose two parts have *r* and *k* number of nodes. The corresponding adjacency matrix can then be represented as the biadjacency matrix [54]

1 $$\begin{aligned} A = \left[ {\begin{array}{*{20}{c}} 0&{}\quad B\\ {{B^T}}&{}\quad 0 \end{array}} \right] . \end{aligned}$$

The link prediction problem is then defined as predicting the unobserved link between two nodes during the time interval $t + \Delta t$. Most of the existing methods solve the link prediction problem by calculating a likelihood score between two nodes (${S_{ij}}$). To validate the models’ performance, we randomly select links from the test set, ${E^T} = E - {E^P}$ and use the remaining set as a training set. The training and test sets are mutually exclusive, i.e., ${E^T} \cap {E^P} = \Phi$.

In this section, we present the link prediction techniques for drug discovery. Our initial steps included the use of existing models meant to solve problems in other domains like social networks. The various models we considered, both old and new, are as follows:

1. Common Neighbours (CN): Two nodes are more likely to be connected if they have more common neighbours. If $\Gamma (i)$ represents the vector associated with node *i* that includes the neighbours of node *i*, we express this relationship as [55]
  2 $$\begin{aligned} {S_{ij}} = |\Gamma (i) \cap \Gamma (j)|. \end{aligned}$$
2. Salton index (Cosine similarity): This is another measure of commonality that measures the cosine of the angle between two vectors of the adjacency matrix, corresponding to given nodes *i* and *j*. It is calculated as below:
  3 $$\begin{aligned} S_{ij} = \frac{{|\Gamma (i) \cap \Gamma (j)\mathrm{{|}}}}{{\sqrt{{k_i} * {k_j}} }}. \end{aligned}$$
  where ${k_i}$ is the total degree of node *i* [56].
3. Jaccard index [57]: This method from the early 20th century is the proportion of common neighbours between two nodes *i* and *j* in the total number of neigbours
  4 $$\begin{aligned} {S_{ij}} = \frac{{|\Gamma (i) \cap \Gamma (j)\mathrm{{|}}}}{{|\Gamma (i) \cup \Gamma (j)|}}. \end{aligned}$$
4. Sorensen index [58]: This index was developed especially for ecological community data and is defined as
  5 $$\begin{aligned} {S_{ij}} = \frac{{2 \times |\Gamma (i) \cap \Gamma (j)\mathrm{{|}}}}{{{k_i} + {k_j}}}. \end{aligned}$$
5. Hub Promoted Index (HPI) [59]: This index was developed for considering metabolic networks. Under this model, links adjacent to hub nodes (high degree nodes) are assigned a high score, as the denominator depends on the minimum of the degrees of the two nodes. It is defined as
  6 $$\begin{aligned} {S_{ij}} = \frac{{|\Gamma (i) \cap \Gamma (j)\mathrm{{|}}}}{{min{{k_i},{k_j}}}}. \end{aligned}$$
6. Hub Depressed Index (HDI) [60]: This index is similar to the Sorensen index, but it also considers the measurement of the opposite effect:
  7 $$\begin{aligned} {S_{ij}} = \frac{{|\Gamma (i) \cap \Gamma (j)\mathrm{{|}}}}{{\max \{ {k_i},{k_j}\} }}. \end{aligned}$$
7. Leicht–Holme–Newman Index (LHN-I) [11]: This model assigns a high score to common neighbour nodes while penalizing according to the degree of each node:
  8 $$\begin{aligned} {S_{ij}} = \frac{{|\Gamma (i) \cap \Gamma (j)|}}{{{k_i}.{k_j}}}. \end{aligned}$$
8. Preferential Attachment (PA) [61]: This is based on the assumption that nodes with higher links will form more links. Therefore, the PA-based link prediction model is simply a product of the degree of the two nodes:
  9 $$\begin{aligned} S_{ij} = {k_i}\cdot {k_j}. \end{aligned}$$
9. Adamic-Adar (AA) [62]: AA uses the assumption that less-connected nodes should be given more weight for future link prediction:
  10 $$\begin{aligned} {S_{ij}} = \sum \limits _{z \in \Gamma (i) \cap \Gamma (j)} {\frac{1}{{\log {k_z}}}}. \end{aligned}$$
10. Resource Allocation Index (RA)[63]: This is inspired by the resource allocation process, and measures how much resource is transmitted between th etwo nodes *i* and *j*:
  11 $$\begin{aligned} {S_{ij}} = \sum \limits _{z \in \Gamma (i) \cap \Gamma (j)} {\frac{1}{{{k_z}}}} \end{aligned}$$
11. Local Path Index (LP) [64, 65]: This index considers the local paths between the two nodes considering neighbours of second order, which makes it fairly inexpensive to compute. For the adjacency matrix *A* of the network, the LP-based score is
  12 $$\begin{aligned} S_{ij}^{LP} = {A^2} + \in {A^3}. \end{aligned}$$
  where $\epsilon$ is a free parameter and when 0, the LP index is equal to *CN*. ${\left( {{A^n}} \right) _{ij}}$ representing the number of paths of length 3 between nodes *i* and *j*.
12. Katz global path indicator [66]: Theis index considers the number of paths, their lengths, and their weights (shorter paths counting more heavily), which is computed as:
  13 $$\begin{aligned} {S_{ij}} = \sum \limits _{l = 1}^\infty {{\beta ^l}} \left| {paths_{ij}^l} \right| = \beta {A_{ij}} + {\beta ^2}{\left( {A^2}\right) _{ij}} + {\beta ^3}{\left( {A^3}\right) _{ij}}.. = {\left( {I - \beta A} \right) ^{ - 1}} - I \end{aligned}$$
  where ${paths_{ij}^l}$ is set of all paths with lenght *l* which connects node *i* and node *j*, $\beta$ is the weight attenuation factor. In order to ensure the convergence of the series, the value of $\beta$ must be less than the reciprocal of the largest eigenvalue of the adjacency matrix *A*.
13. Average Commute Time (ACT) [67]: This metric determines closeness by commute time. The smaller the average commute time between the two nodes, the closer the nodes are. It considers the average number of steps required by a random walker starting from *i* to reach *j* and vice versa. It can be calculated as:
  14 $$\begin{aligned} S_{ij}^{ACT}= & {} \frac{1}{{l_{ii}^ + + l_{jj}^ + - 2l_{ij}^ + }}\quad \quad \begin{array}{l} {v^i} = \sqrt{\Lambda }\cdot {U^T}{\overset{\rightharpoonup }{e}}_{i},\\ {l_{ij}}^ + = {v_i}^T{v_j}, \end{array} \end{aligned}$$
  Where $l_{ij}^ +$ entry of Laplacian matrix $L=D-A$. Where *D* is the degree matrix.
14. Cosine Similarity Based on Random Walk (Cos+) [68]: This method uses the inner product. Letting $e^i$ be the $N\times 1$ vector with the *ith* entry$=1$, then
  15 $$\begin{aligned} {v^i} = \sqrt{\Lambda }\cdot {U^T}{\overset{\rightharpoonup }{e}_i}. \end{aligned}$$
  If *U* is the orthonormal matrix made of eigenvectors of the Laplacian matrix *L*, $\Lambda =$ diag($\lambda _{i}$), and
  16 $$\begin{aligned} S_{ij}^{\cos + } = \cos {(i,j)^ + } = \frac{{l_{ij}^ + }}{{\sqrt{l_{ii}^ + l_{jj}^ + } }}. \end{aligned}$$
  where symbols have usual meaning.
15. Random Walk with Restart (RWR)[69]: This is inspired by the Google PageRank algorithm. Suppose a random walker at node *i* takes a random step towards any of the neighbours of *i* with probability $\alpha$. Therefore the probability of returning to node *i* again is ($1-\alpha$). Thus, the probability that the random walker reaches node *j* can be given as ${\vec{P}_i} = \alpha {L^T}\vec{P} + (1 - \alpha {L^T}){e^i}$, where $L^T$ is the transition matrix ${\mathrm{{L}}_{ij}}\mathrm{{ = }}\frac{\mathrm{{1}}}{{{k_i}}}$ if node *i* and *j* are connected other wise ${L}_{ij}=0$ .
  17 $$\begin{aligned} S_{ij}^{RWR} = {P_{ij}}+{P_{ji}}, \end{aligned}$$
  where $p_{ij}$ is the *jth* element of vector $p_i$.
16. Local Random Walk (LRW) [70]: A random walker starts at node *i* and reaches node *j* within some number of random steps. The initial density vector is $\overrightarrow{{\Pi _{(0)}}} = \overrightarrow{{e_i}}$. The LRW similarity index at any time *t* can be formulated as
  18 $$\begin{aligned} S_{ij}^{LRW}(t) = {P_i}\Pi _{ij}^{}(t) + {P_j}{\Pi _{ji}}(t), \end{aligned}$$
  where *P* is the initial configuration function.
17. SimRank (SimR) [71]: This is a general similarity measure that considers two nodes are similar if their neighbours are similar (connected to similar nodes):
  19 $$\begin{aligned} s_{ij}^{SimR} = C\frac{{\sum \nolimits _{z \in \Gamma (i)} {\sum \nolimits _{z' \in \Gamma (j)} {S_{zz'}^{SimR}} } }}{{{k_i}{k_j}}}. \end{aligned}$$
18. CN based on Transferring Similarity (TSCN) [72]: This method uses the CN index but considers the transference similarity deined as
  20 $$\begin{aligned} S_{ij}^{Tr} = \varepsilon \sum \limits _v {S_{ij}^{CN}S_{vj}^{Tr}} + S_{ij}^{CN}, \end{aligned}$$
  where $S_{vj}^{Tr}$ is the transferring similarity.
19. Superimposed Local Random Walk Indicator (SRW) [70]: This method is based on LRW, summing the *t* step and its previous results to obtain the value of SRW:
  21 $$\begin{aligned} S_{ij}^{SRW}(t) = \sum \limits _{l = 1}^t {S_{ij}^{LRW}} (l) = {q_i}\sum \limits _{}^t {{\pi _{ij}}(l)} + {q_j}\sum \limits _{l = 1}^t {{\pi _{ji}}(l)}. \end{aligned}$$
20. Local Naive Bayes form of CN (LNBCN) [73]:
  22 $$\begin{aligned} S_{ij}^{LNBRA} = \sum \limits _{w \in \Gamma (i) \cap \Gamma (j)} {\frac{1}{{{k_w}}}} (\log {R_w} + \log S), \end{aligned}$$
  where $S = \frac{{P({A_0})}}{{P({A_1})}}$, and ${A_0}$ and $A_1$ are the connection and disconnection variables, respectively. $R_W$ is the role function of node *w*.
21. Local Naive Bayes form of RA (LNBRA) [59]: This method is based on Naive Bayes and formulated as follows
  23 $$\begin{aligned} S_{ij}^{LNBRA} = \left| {\Gamma (i) \cap \Gamma (j)} \right| \log s + \sum \limits _{w \in \Gamma (i) \cap \Gamma (j)} {\log {R_w}}, \end{aligned}$$
  where $S = \frac{{P({A_0})}}{{P({A_1})}}$, and $A_0$ and $A_1$ are the connection and disconnection variables, respectively. $R_W$ is the role function of node *w*.
22. Leicht–Holme–Newman (LHN2) Index [11]: This index is based on the assumption that two nodes are similar if their neighbours are also similar. It is an extension of the Katz index. The LHN2 likelihood score can be given as
  24 $$\begin{aligned} S_{ij}^{LHN2} = 2m{\lambda _1}{D^{ - 1}}{\left( I - \frac{{\Phi A}}{{{\lambda _1}}}\right) ^{ - 1}}{D^{ - 1}}, \end{aligned}$$
  where *D* is the degree matrix with ${D_{ij}} = {\lambda _{ij}}{k_i}$, and $\Phi \varepsilon (0,1)$ is the free parameter.
23. Cosine based on L+ (CosPlus): This similarity measure is based on the inner product measure and cosine similarity between node vectors *i* and *j*. It is given as
  25 $$\begin{aligned} S_{ij}^{Cos + } = \frac{{V_i^T\cdot V_i^T}}{{\left| {{V_i}} \right| \cdot \left| {{V_j}} \right| }}. \end{aligned}$$
24. Matrix Forest Index (MFI) [74]: The MFI index similarity between nodes *i* and *j* can be given as ratio of the number of spanning rooted forests so that nodes *i* and *j* belong to the same tree rooted at *i* to all spanning rooted forests. This similarity index is expressed as
  26 $$\begin{aligned} S_{ij}^{MFI} = {(I + L)^{ - 1}}. \end{aligned}$$
25. Prone [75]: This method first initialises the embedding by sparse matrix factorization and further uses spectral analysis for local and global structural information of the node.
26. DeepWalk [76]: This model learns node low dimensional embeddings based on random walks. It has two hyper parameters: the walk length *l* and the window size *w*.
27. Node2vec [77]: Node2vec is an application of the Word2vec model for graphs [78]. Word2vec is a state-of-the-art framework for word embedding. Based on similar skip-gram concept Node2vec works on neighbourhood nodes and generates low dimensional embeddings. Node2vec can be generalised according to need, such as if one wants to embed similarity based on distance or based on role of the node in network.
28. LINE [79]: This model generates low-level node embeddings considering first order and second order of the nodes’ similarity. Further, this model samples based on edge weight, improving performance for large scale networks. It is special case of DeepWalk when the size of the vertices’ context is kept at 1.
29. NetMF [80]: Similar to DeepWalk and Line, this method also employs the skip-gram technique for low dimensional embedding. In fact, this model unifies the LINE, PTE [81], DeepWalk, Node2vec, and the proposed matrix factorization framework.
30. High-Order Proximity-preserved Embedding (HOPE) [82]: This method draws from PageRank and the Katz index and uses singular value decomposition for making low rank approximations.
31. NetSMF [80]: Network Embedding as Sparse Matrix Factorization (NetSMF) is based on spectral sparsification, and is an improved extension of NetMF. It is costly for large networks as it requires a large number of random walks.
32. GraRep [83]: Grarep depends on singular value decomposition. It uses nodes’ co-occurrence information by exponentiating the matrix with different powers, making it unsuitable for large graphs.

### Model performance evaluation metrics

We convert the link prediction problem to a binary classification problem by using a positive class from the test set ($E^T$). Further, we generate negative samples for training and test sets. To sample negative links for training and test data set, we assume all the testing links were known, and thus sample negative train links only from other unknown links. This enables us to evaluate the accuracy of link prediction methods based on binary classification evaluation metrics. To evaluate performance we used three standard machine learning metrics as follows:

*Precision* Precision measures the proportion of true positives against all positives. For $T_P$ items predicted correctly as positive and $F_P$ are predicted incorrectly as positive (i.e., false positives). Precision is calculated as:

27 $$\begin{aligned} {\mathrm{Precision}} = \frac{{{T_P}}}{{{T_P} + {F_P}}}\ . \end{aligned}$$

To measure the misclassification of actual positives, we use the Recall metric, penalising the score with false negatives. If $F_N$ is the number of false negatives, then recall is defined as

28 $$\begin{aligned} {{Recall}} = \frac{{{T_P}}}{{{T_P} + {F_N}}}. \end{aligned}$$

Finally, a combined scoring mechanism called the F1-Score is a harmonic mean between precision and recall. It is also known as the True Positive Rate (TPR):

29 $$\begin{aligned} F1{-}score= \frac{{{\mathrm{Precision}} *{\mathrm{Recall}} }}{{\left( {\mathrm{Precision}} + {\mathrm{Recall}} \right) }} \end{aligned}$$

The False Positive Rate (FPR) is calculated as

30 $$\begin{aligned} FPR = \frac{{{F_P}}}{{\left( {T_N} + {F_P}\right) }}, \end{aligned}$$

where $F_P$ is the number of false positives, and $T_N$ is the number of true negatives.

*AUROC* The Area Under the Receiver Operating Characteristics (AUROC) value is the area under the plot between True Positive Rate (TPR) and the False Positive Rate (FPR). It represents the trade-off between TP and FP prediction rates. The TPR is also known as sensitivity, recall, or probability of detection. AUROC measures the separability of the classifier and is therefore a vital metric.

*AUPR* The area under the Precision and Recall (AUPR) curve estimates the combined accuracy of precision and recall simultaneously. In other words, precision–recall pair points are obtained by considering different threshold values. This measure estimates the efficiency in the presence of unbalanced classes and indicates the models’ ability to cope with skewed distributions.

### Datasets used

We used several publicly available datasets for pharmacological problems:

- Disease–Gene Association network (DGA): This is a disease-gene association network dataset from the Stanford SNAP group and contains disease and gene association information [84].
- Drug–Disease Association network (DDA): There are two kinds of nodes in this network: drugs and diseases. Some nodes in the drugs class are non-pharmaceutical chemicals such as arsenic. Diseases include skin disease and myocardial infarction. These interactions predict which drug treats which diseases.
- Disease–Target Interaction network (DTI): This source is from the Stanford SNAP online data repository with DTI information similar to MATADOR. We use the protein and chemical identifiers as the two kinds of nodes in a bipartite network [85].
- MATADOR database: This is a manually annotated drug and target database freely available [86] and containing 15,843 Drug–Target interactions (DTIs). The original data set contains 13 fields, but in this study we only used two as a sample: chemical identifier, and protein identifier.
- Drug–Drug Interaction network (DDI): This source is also from the Stanford SNAP group, containing information about drug–drug interactions approved by the United States Food and Drug Administration. It is a mono-partite network as compared to the previous bipartite datasets. A drug–drug relationship is formed when the pharmacological effect of one drug is affected by another drug [85].

The Table 1 summarises the contents of the datasets.

**Table 1:** Some properties of the data sets used in our experiments

Dataset	|*V*|	|*E*|	From nodes	To nodes
MATADOR	3702	15,843	801	2901
DTI	3932	18,690	284	3648
DDI	1514	48,514	1514	1514
DGA	7813	21,357	519	729
DDA	7197	466,656	5535	1662

## Analysis and results

As cross-validation is a standard technique to test the generalizing ability of models or algorithms, we performed a 10-fold cross-validation. We randomly selected a percentage of edges and removed them from the network, and used those removed edges as test data in each model. Using these two sets, we then evaluated the performance of the models. Table 2 shows the results, where the best results are highlighted in each case. For several models we considered a variation of hyper-parameters which gives better results such as random walk length etc. For instance, the numbers after LRW model name (i.e. $LRW_3$, $LRW_4$, $LRW_5$) reflects the number of random-walk steps. We make the following points in our own analysis:

- On the Disease-Gene associate (DGA) dataset, the Average Commute Time (ACT) model achieved the best AUROC score, the $LRW_3$ (Local Random Walk) model with 3 steps achieved the best AUPR score, and the LHN2 (Leicht–Holme–Newman) with parameter 0.95 achieved the best F1-score. All the three models are considering global similarity measures.
- On the Drug-Disease association (DDA) dataset, again the ACT achieved the best AUROC score, LRW with 5 steps achieved the best AUPR score, and LHN2 with parameter 0.95 had the best F1-score.
- On the Disease–Target Interaction (DTI) dataset, NetMF performed the best on all three metric scores.
- On the MATADOR dataset, NetMF performed best on all three metrics.
- On the Drug–Drug Interaction (DDI) dataset, as the only mono-partite network, the Prone model performed best on all three metrics.

Overall, the models that performed best on the five benchmark datasets were *Prone*, *ACT* and $LRW_5$ are the top 3. The Freidman test results are presented in Table 3.

**Table 2:** Model performance according to the three evaluation metrics: AUROC, AUPR, and F1-score

Datasets	DGA			DDA			DTI			Matador			DDI		
Models	AUROC	AUPR	F1	AUROC	AUPR	F1	AUROC	AUPR	F1	AUROC	AUPR	F1	AUROC	AUPR	F1
CN	0.5733	0.7803	0.5	0.5733	0.7803	0.5	0.5733	0.7803	0.5	0.5733	0.7803	0.5	0.5733	0.7803	0.5
Salton	0.5732	0.7776	0.5	0.5732	0.7776	0.5	0.5732	0.7776	0.5	0.5732	0.7776	0.5	0.5732	0.7776	0.5
Jaccard	0.5732	0.7765	0.5	0.5732	0.7765	0.5	0.5732	0.7765	0.5	0.5732	0.7765	0.5	0.5732	0.7765	0.5
Sorensen	0.5732	0.7765	0.5	0.5732	0.7765	0.5	0.5732	0.7765	0.5	0.5732	0.7765	0.5	0.5732	0.7765	0.5
HPI	0.5733	0.7265	0.5	0.5733	0.7265	0.5	0.5733	0.7265	0.5	0.5733	0.7265	0.5	0.5733	0.7265	0.5
HDI	0.5732	0.7765	0.5	0.5732	0.7765	0.5	0.5732	0.7765	0.5	0.5732	0.7765	0.5	0.5732	0.7765	0.5
LHN	0.5732	0.7773	0.5	0.5732	0.7773	0.5	0.5732	0.7773	0.5	0.5732	0.7773	0.5	0.5732	0.7773	0.5
AA	0.5733	0.7807	0.5	0.5733	0.7807	0.5	0.5733	0.7807	0.5	0.5733	0.7807	0.5	0.5733	0.7807	0.5
RA	0.5733	0.7805	0.5	0.5733	0.7805	0.5	0.5733	0.7805	0.5	0.5733	0.7805	0.5	0.5733	0.7805	0.5
PA	0.451	0.5183	0.5	0.451	0.5183	0.5	0.451	0.5183	0.5	0.451	0.5183	0.5	0.451	0.5183	0.5
LNBCN	0.5733	0.7811	0.5	0.5733	0.7811	0.5	0.5733	0.7811	0.5	0.5733	0.7811	0.5	0.5733	0.7811	0.5
LNBAA	0.5733	0.7809	0.5	0.5733	0.7809	0.5	0.5733	0.7809	0.5	0.5733	0.7809	0.5	0.5733	0.7809	0.5
LNBRA	0.5733	0.7808	0.5	0.5733	0.7808	0.5	0.5733	0.7808	0.5	0.5733	0.7808	0.5	0.5733	0.7808	0.5
LocalP	0.6133	0.8004	0.5	0.6133	0.8004	0.5	0.6133	0.8004	0.5	0.6133	0.8004	0.5	0.6133	0.8004	0.5
Katz .01	0.596	0.728	0.5	0.596	0.728	0.5	0.596	0.728	0.5	0.596	0.728	0.5	0.596	0.728	0.5
$\sim .001$	0.596	0.728	0.5	0.596	0.728	0.5	0.596	0.728	0.5	0.596	0.728	0.5	0.596	0.728	0.5
LHN2 .9	0.596	0.728	0.5	0.596	0.728	0.5	0.596	0.728	0.5	0.596	0.728	0.5	0.596	0.728	0.5
$\sim .95$	0.6488	0.7606	0.7492	0.6488	0.7606	0.7492	0.6488	0.7606	0.7492	0.6488	0.7606	0.7492	0.6488	0.7606	0.7492
$\sim .99$	0.6486	0.76	0.7481	0.6486	0.76	0.7481	0.6486	0.76	0.7481	0.6486	0.76	0.7481	0.6486	0.76	0.7481
ACT	0.8134	0.7868	0.7272	0.8134	0.7868	0.7272	0.8134	0.7868	0.7272	0.8134	0.7868	0.7272	0.8134	0.7868	0.7272
CosPlus	0.6975	0.7409	0.7192	0.6975	0.7409	0.7192	0.6975	0.7409	0.7192	0.6975	0.7409	0.7192	0.6975	0.7409	0.7192
RWR .85	0.5988	0.7321	0.5	0.5988	0.7321	0.5	0.5988	0.7321	0.5	0.5988	0.7321	0.5	0.5988	0.7321	0.5
$\sim .95$	0.5994	0.7331	0.5	0.5994	0.7331	0.5	0.5994	0.7331	0.5	0.5994	0.7331	0.5	0.5994	0.7331	0.5
SimR	0.7019	0.8202	0.5	0.7019	0.8202	0.5	0.7019	0.8202	0.5	0.7019	0.8202	0.5	0.7019	0.8202	0.5
LRW 3	0.6607	0.8054	0.4462	0.6607	0.8054	0.4462	0.6607	0.8054	0.4462	0.6607	0.8054	0.4462	0.6607	0.8054	0.4462
$\sim 4$	0.7033	0.8255	0.4462	0.7033	0.8255	0.4462	0.7033	0.8255	0.4462	0.7033	0.8255	0.4462	0.7033	0.8255	0.4462
$\sim 5$	0.7376	0.8443	0.7335	0.7376	0.8443	0.7335	0.7376	0.8443	0.7335	0.7376	0.8443	0.7335	0.7376	0.8443	0.7335
SRW 3	0.6606	0.8042	0.4462	0.6606	0.8042	0.4462	0.6606	0.8042	0.4462	0.6606	0.8042	0.4462	0.6606	0.8042	0.4462
$\sim 4$	0.7034	0.8261	0.4462	0.7034	0.8261	0.4462	0.7034	0.8261	0.4462	0.7034	0.8261	0.4462	0.7034	0.8261	0.4462
$\sim 5$	0.7376	0.8432	0.7334	0.7376	0.8432	0.7334	0.7376	0.8432	0.7334	0.7376	0.8432	0.7334	0.7376	0.8432	0.7334
MFI	0.5978	0.7302	0.5	0.5978	0.7302	0.5	0.5978	0.7302	0.5	0.5978	0.7302	0.5	0.5978	0.7302	0.5
TSCN	0.5906	0.7196	0.5	0.5906	0.7196	0.5	0.5906	0.7196	0.5	0.5906	0.7196	0.5	0.5906	0.7196	0.5
Prone	0.7389	0.6995	0.647	0.7864	0.6803	0.7269	0.8373	0.8129	0.7158	0.9486	0.9346	0.9063	0.9405	0.9108	0.8854
NetMF	0.7548	0.7262	0.692	0.4916	0.5078	0.5156	0.8879	0.8826	0.8182	0.955	0.941	0.9238	0.854	0.8435	0.8025
Node2vec	0.5898	0.5621	0.5153	0.5819	0.5175	0.5646	0.6973	0.6457	0.5543	0.9157	0.8864	0.8526	0.8065	0.7876	0.7357
Deepwalk	0.5871	0.5592	0.5098	0.5805	0.516	0.5657	0.698	0.6463	0.5548	0.9151	0.8863	0.8491	0.8057	0.7869	0.7336
Hope	0.6304	0.5313	0.5542	0.6086	0.5542	0.6075	0.7318	0.6172	0.6128	0.8304	0.7457	0.7841	0.8894	0.866	0.8076
NetSMF	0.5721	0.5282	0.5153	0.3822	0.3994	0.4111	0.7191	0.6449	0.5987	0.7923	0.7191	0.7431	0.7095	0.6736	0.6733
Grarep	0.0724	0.277	0	0.2765	0.3671	0.318	0.3796	0.3252	0.1624	0.2992	0.3522	0.2538	0.6739	0.6286	0.6435

### Advatanges and disadvantages of these methods

No doubt the graphs are state-of-the art tools being utilised to solve problems of complex systems. But as they give strength to model any real world problem they also have weaknesses specially in our case. As graphs are representations of nodes and edges. A node alone carries less or no information at all. The node of the graph carries more information if it is the part of bigger networks or graphs. Consequently our model will perform worse for node/s which are either alone or connected to small subgraphs. For example in case of drug–drug side effect prediction if we have more examples of side-effect interaction examples with other drugs then the unknown interaction can be predicted with better accuracy. The same logic goes with drug-target interaction prediction and so on. Consequently we can say the more edges we have the better the predictibility of a model. So network based methods will fail to predict interaction for novel node/s which has no prior interaction say it is new drug or new target.

### Statistical test

After running the Friedman test [87] for model comparison we have found the following mean ranking of models’ across the datasets. To achieve this we have only considered one evaluation metric i.e. *AUROC* and converted into error using formula $AUROC_{error}=1-AUROC$. According to Table 3 analysis we can say on an average *Prone*, *ACT* and $LRW_5$ are the top 3 best performers over all five data-sets. The p-value is found $1.11022E-16$. Rest of the tests we have reported in Additional file 1.

**Table 3:** Average performance of all models across datasets

Model	CN	Salton	Jaccard	Sorens	HPI	HDI	LHN	AA	RA	PA
Mean rank	28.6	34.6	34.6	34.6	28.6	34.6	34.6	28.6	28.6	38
Model	LNBCN	LNBAA	LNBRA	LocalP	Katz.01	$\sim$.001	LHNII.9	$\sim$.95	$\sim$.99	ACT
Mean rank	28.6	28.6	28.6	16.6	21.8	21.8	21.8	14.4	15.4	3

Model	CosPlus	RWR.85	$\sim$.95	SimR	LRW_3	$\sim$_4	$\sim$_5	SRW_3	$\sim$_4	$\sim$_5
Mean rank	11	18.8	17.8	9.8	12.4	8.8	5.7	13.4	7.8	5.7
Model	MFI	TSCN	prone	netmf	node2vec	Deepwalk	hope	netsmf	grarep	
Mean rank	19.8	23.8	2	8.6	13.2	13.6	8.2	19.6	34	

## Conclusion and discussion

This study highlighted the need for utilising data-driven approaches for enhancing drug discovery processes particularly using the drug-target interactions (DTI) forming biomedical networks. This allows us to utilise network-based techniques and in particular link prediction approaches to predict the interactions (links), or missing links, between drugs and their targets including diseases, proteins or other drugs. This approach has been already shown promising outcomes in reusing existing drugs for treating breast cancer [14], or identifying a new drug for urine leakage issues treatment [16]. We further discussed more advanced network-based approaches are required to addresses the existing challenges in using traditional machine learning approaches such as data related issues (e.g. dimensionality, incompleteness, sparsity, heterogeneity, and the hierarchical structure), and incapability of consideration of the spreading speed of diseases.

In this work, we have compared several state-of-the-art link prediction models on five different drug-related data-sets modeling drug–disease, drug–drug, drug–gene, and drug–target interactions to see the implications for drug discovery. We compared the results of the models using three evaluation metrics namely AUROC, AUPR, and F1-score. The results indicate that *Prone*, *ACT* and $LRW_5$ are the top 3 best performers on all five data-sets. These models are important as they only need prior link or relationship information, which avoids the cost of feature engineering. The statistical models are effective particularly on graphs as the graph is basically a non-Euclidean data representation. Traditional machine learning tools were intended for Euclidean data sets, thus explaining the performance. There are excellent opportunities for future work to represent and solve network-based biological and pharmaceutical problems using state-of-the-art deep learning techniques.

### Methodological limitations

In our analysis we have considered positive links only from examples we had in the data-set. Rest of the space is considered as negative links which is acceptable for mathematical algorithmic perspective. This techniques is well utilised by machine learning community. The negative link space is all the unknowns for which we don’t have any examples. So one of the limitations for our methodology is that if we have lesser number of examples the algorithmic predictive ability will be negatively affected. In other words the more examples we have the better the predictability of the algorithm will be.

## Supplementary Information

**Additional file 1:** Data normality test.

## Data Availability

The data-sets can be found at https://github.com/khushnood/DataDrivenDrugDiscovery/tree/master.

## Abbreviations

AUROC: The Area Under the Receiver Operating Characteristics curve
AUPR: The area under the Precision and Recall curve
TPR: True positive rate
FPR: False positive rate
FDA: Food and Drug Administration
ML: Machine learning
DTI: Drug–target interactions
TBSI: Target based similarity inference
NBI: Network based inference
NRWRH: Network-based Random Walk with Restart on the Heterogeneous network
DSESI: Drug side effect similarity inference
MTOI: Multiple target optimal intervention
GCPN: Graph Convolutional Policy Network
GAN: Generative Adversarial Network
LINCS: Library of integrated network-based cellular signatures
SIDER: Side effect resource
ligENTS: Ligand Enrichment of Network Topological Similarity
DLBCL: Diffuse large B-cell lymphoma
ADR: Adverse Drug Reactions
BFRM: Bayesian Factor Regression Model
CSB: Cancer Signalling Bridges
CN: Common Neighbours
HPI: Hub Promoted Index
HDI: Hub Depressed Index
LHNI: Leicht–Holme–Newman Index
PA: Preferential Attachment
AA: Adamic-Adar
RAI: Resource Allocation Index
LPI: Local Path Index
ACT: Average Commute Time
RWR: Random Walk with Restart
RWRn: Random Walk with Restart with n steps
LRW: Local Random Walk
LRWn: Local Random Walk with n steps
TSCN: CN based on Transferring Similarity
SLRWI: Superimposed Local Random Walk Indicator
LNBCN: Local Naive Bayes form of CN
LNBRA: Local Naive Bayes form of RA
LHN2: Leicht–Holme–Newman
MFI: Matrix Forest Index
LINE: Large-scale Information Network Embedding
HOPE: High-Order Proximity-preserved Embedding
NetSMP: Network Embedding as Sparse Matrix Factorization
Prone: A graph vertex representation model name
GraRep: A graph vertex representation model name
DeepWalk: A graph vertex representation model name
Node2Vec: A graph vertex representation model name
DGA: Disease-Gene Association network
DDA: Drug-Disease Association network
DTI: Disease–Target Interaction network
DDI: Drug–Drug Interaction network
MATADOR: Protein–chemical interactions
