# Supplementary material for: Application of network link prediction in drug discovery
Source: BMC Bioinformatics. 2021 Apr 12;22:187. doi: 10.1186/s12859-021-04082-y (PMC8042985; doi:10.1186/s12859-021-04082-y)
Supplement: Supplementary file 1 — Additional file 1: Data normality test. [file 12859_2021_4082_MOESM1_ESM.pdf]

Application of network link prediction in drug discovery: supplemental document

This is supplementary information for "Application of network link prediction in drug discovery".

1. SHAPHIRO-WILK NORMALITY TESTS OF THE RESULTS

Here we presents the normality tests report of Shaphiro-wilk [1] tests results. We have presented the p-value of our normality tests of 10 generated results.

Table S1. P-value of the normality tests of our 10-fold cross validation results.

Model	DGA	DDA	Matadoor	DTI	DDI
CN	0.79481	0.70266	0.90985	0.21523	0.39505
Salton	0.06959	0.45454	0.92826	0.68971	0.40904
Jaccard	0.8308	0.44919	0.12941	0.81832	0.08995
Sorens	0.86372	0.83481	0.01473	0.51624	0.82011
HPI	0.52056	0.22235	0.80214	0.52167	0.70653
HDI	0.02085	0.35159	0.07789	0.40331	0.81069
LHN	0.12232	0.47553	0.01413	0.03343	0.47661
AA	0.21967	0.76986	0.02484	0.64768	0.14119
RA	0.46554	0.58525	0.52285	0.72483	0.86221
PA	0.00515	0.90991	0.85119	0.38271	0.01209
LNBCN	0.1942	0.01096	0.71814	0.42578	0.25423
LNBAA	0.07586	0.20439	0.79121	0.20813	0.96554
LNBRA	0.05343	0.05988	0.61907	0.8301	0.05251
LocalP	0.22281	0.88416	0.5855	0.11142	0.26086
Katz.01	0.27752	0.6411	0.30219	0.98171	0.71915
∼.001	0.01077	0.03839	0.46608	0.6542	0.6043
LHNII.9	0.01077	0.03839	0.46608	0.6542	0.6043
∼.95	0.01077	0.03839	0.46608	0.6542	0.6043
∼.99	0.01077	0.03839	0.46608	0.6542	0.6043
ACT	0.32746	0.86959	0.60445	0.83174	0.62093
CosPlus	0.89245	0.42556	0.30313	0.81287	0.81377
RWR.85	0.54166	0.06524	0.52404	0.71435	0.69588
∼.95	0.22724	0.30996	0.84251	0.53375	0.57231
SimR	0.22724	0.30996	0.84251	0.53375	0.57231
LRW_3	0.5917	0.68159	0.61714	0.09497	0.81485
∼_4	0.78782	0.72649	0.32267	0.39003	0.28578

Table S1. P-value of the normality tests of our 10-fold cross validation results.

Model	DGA	DDA	Matadoor	DTI	DDI
∼_5	0.7163	0.59284	0.07955	0.09415	0.53866
SRW_3	0.27078	0.06993	0.68071	0.17221	0.80954
∼_4	0.754	0.68743	0.69839	0.13357	0.02081
∼_5	0.55365	0.44357	0.60222	0.79945	0.54719
MFI	0.76882	0.86222	0.46362	0.88972	0.21698
TSCN	0.64941	0.3489	0.15333	0.46545	0.88327
Prone	0.06113	0.06339	0.21471	0.02583	0.2622
NetMF	0.01209	0.02056	0.2388	0.24808	0.1351
Node2Vec	0.32345	0.04977	0.54069	0.7396	0.1351
DeepWalk	0.01785	0.3053	0.25771	0.11561	0.27543
Hope	0.491639	0.02938	0.49266	0.54069	0.11561
NetSMF	0.4132	0.7881	0.35329	0.36211	0.353294
Grarep	0.85945	0.87028	0.98565	0.67028	0.933567

2. MODEL PERFORMANCE TESTS

Here we present overall error score of all the models on the basis of average score on all the five data-sets. The result is generated based on Friedman test[2].

Table S2. This table presents the average error score ($AUROC_{error}$)of all the models ac cross all the datasets.

Model	Min	Q1	Median	Q3	Max
CN	0.4267	0.4267	0.4267	0.4267	0.4267
Salton	0.4268	0.4268	0.4268	0.4268	0.4268
Jaccard	0.4268	0.4268	0.4268	0.4268	0.4268
Sorens	0.4268	0.4268	0.4268	0.4268	0.4268
HPI	0.4267	0.4267	0.4267	0.4267	0.4267
HDI	0.4268	0.4268	0.4268	0.4268	0.4268
LHN	0.4268	0.4268	0.4268	0.4268	0.4268
AA	0.4267	0.4267	0.4267	0.4267	0.4267
RA	0.4267	0.4267	0.4267	0.4267	0.4267
PA	0.549	0.549	0.549	0.549	0.549
LNBCN	0.4267	0.4267	0.4267	0.4267	0.4267
LNBAA	0.4267	0.4267	0.4267	0.4267	0.4267
LNBRA	0.4267	0.4267	0.4267	0.4267	0.4267
LocalP	0.3867	0.3867	0.3867	0.3867	0.3867
Katz.01	0.404	0.404	0.404	0.404	0.404
∼.001	0.404	0.404	0.404	0.404	0.404

Table S2. This table presents the average error score ($AUROC_{error}$)of all the models ac cross all the datasets.

Model	Min	Q1	Median	Q3	Max
LHNII.9	0.404	0.404	0.404	0.404	0.404
∼.95	0.3512	0.3512	0.3512	0.3512	0.3512
∼.99	0.3514	0.3514	0.3514	0.3514	0.3514
ACT	0.1866	0.1866	0.1866	0.1866	0.1866
CosPlus	0.3025	0.3025	0.3025	0.3025	0.3025
RWR.85	0.4012	0.4012	0.4012	0.4012	0.4012
∼.95	0.4006	0.4006	0.4006	0.4006	0.4006
SimR	0.2981	0.2981	0.2981	0.2981	0.2981
LRW_3	0.3393	0.3393	0.3393	0.3393	0.3393
∼_4	0.2967	0.2967	0.2967	0.2967	0.2967
∼_5	0.2624	0.2624	0.2624	0.2624	0.2624
SRW_3	0.3394	0.3394	0.3394	0.3394	0.3394
∼_4	0.2966	0.2966	0.2966	0.2966	0.2966
∼_5	0.2624	0.2624	0.2624	0.2624	0.2624
MFI	0.4022	0.4022	0.4022	0.4022	0.4022
TSCN	0.4094	0.4094	0.4094	0.4094	0.4094
prone	0.0514	0.05545	0.1627	0.23735	0.2611
netmf	0.045	0.07855	0.146	0.3768	0.5084
node2vec	0.0843	0.1389	0.3027	0.41415	0.4181
Deepwalk	0.0849	0.1396	0.302	0.4162	0.4195
hope	0.1106	0.1401	0.2682	0.3805	0.3914
netsmf	0.2077	0.2443	0.2905	0.52285	0.6178
grarep	0.3261	0.47325	0.7008	0.82555	0.9276

3. TEST TABLE

REFERENCES

1. S. S. Shapiro and M. B. Wilk, "An analysis of variance test for normality (complete samples)," Biometrika **52**, 591–611 (1965).
2. M. Friedman, "The use of ranks to avoid the assumption of normality implicit in the analysis of variance," J. american statistical association **32**, 675–701 (1937).

Table S3. Model performance according to the three evaluation metrics: AUROC, AUPR, and F1-score (F1)

**Datasets**	**DGA**			**DDA**			**DTI**			**Matador**			**DDI**		
Models	**AUROC**	**AUPR**	**F1**	**AUROC**	**AUPR**	**F1**	**AUROC**	**AUPR**	**F1**	**AUROC**	**AUPR**	**F1**	**AUROC**	**AUPR**	**F1**
**CN**	0.5733	0.7803	0.5	0.5733	0.7803	0.5	0.5733	0.7803	0.5	0.5733	0.7803	0.5	0.5733	0.7803	0.5
**Salton**	0.5732	0.7776	0.5	0.5732	0.7776	0.5	0.5732	0.7776	0.5	0.5732	0.7776	0.5	0.5732	0.7776	0.5
**Jaccard**	0.5732	0.7765	0.5	0.5732	0.7765	0.5	0.5732	0.7765	0.5	0.5732	0.7765	0.5	0.5732	0.7765	0.5
**Sorensen**	0.5732	0.7765	0.5	0.5732	0.7765	0.5	0.5732	0.7765	0.5	0.5732	0.7765	0.5	0.5732	0.7765	0.5
**HPI**	0.5733	0.7265	0.5	0.5733	0.7265	0.5	0.5733	0.7265	0.5	0.5733	0.7265	0.5	0.5733	0.7265	0.5
**HDI**	0.5732	0.7765	0.5	0.5732	0.7765	0.5	0.5732	0.7765	0.5	0.5732	0.7765	0.5	0.5732	0.7765	0.5
**LHN**	0.5732	0.7773	0.5	0.5732	0.7773	0.5	0.5732	0.7773	0.5	0.5732	0.7773	0.5	0.5732	0.7773	0.5
**AA**	0.5733	0.7807	0.5	0.5733	0.7807	0.5	0.5733	0.7807	0.5	0.5733	0.7807	0.5	0.5733	0.7807	0.5
**RA**	0.5733	0.7805	0.5	0.5733	0.7805	0.5	0.5733	0.7805	0.5	0.5733	0.7805	0.5	0.5733	0.7805	0.5
**PA**	0.451	0.5183	0.5	0.451	0.5183	0.5	0.451	0.5183	0.5	0.451	0.5183	0.5	0.451	0.5183	0.5
**LNBCN**	0.5733	0.7811	0.5	0.5733	0.7811	0.5	0.5733	0.7811	0.5	0.5733	0.7811	0.5	0.5733	0.7811	0.5
**LNBAA**	0.5733	0.7809	0.5	0.5733	0.7809	0.5	0.5733	0.7809	0.5	0.5733	0.7809	0.5	0.5733	0.7809	0.5
**LNBRA**	0.5733	0.7808	0.5	0.5733	0.7808	0.5	0.5733	0.7808	0.5	0.5733	0.7808	0.5	0.5733	0.7808	0.5
**LocalP**	0.6133	0.8004	0.5	0.6133	0.8004	0.5	0.6133	0.8004	0.5	0.6133	0.8004	0.5	0.6133	0.8004	0.5
**Katz .01**	0.596	0.728	0.5	0.596	0.728	0.5	0.596	0.728	0.5	0.596	0.728	0.5	0.596	0.728	0.5
**∼.001**	0.596	0.728	0.5	0.596	0.728	0.5	0.596	0.728	0.5	0.596	0.728	0.5	0.596	0.728	0.5
**LHN2 .9**	0.596	0.728	0.5	0.596	0.728	0.5	0.596	0.728	0.5	0.596	0.728	0.5	0.596	0.728	0.5
**∼.95**	0.6488	0.7606	**0.7492**	0.6488	0.7606	**0.7492**	0.6488	0.7606	0.7492	0.6488	0.7606	0.7492	0.6488	0.7606	0.7492
**∼.99**	0.6486	0.76	0.7481	0.6486	0.76	0.7481	0.6486	0.76	0.7481	0.6486	0.76	0.7481	0.6486	0.76	0.7481
**ACT**	**0.8134**	0.7868	0.7272	**0.8134**	0.7868	0.7272	0.8134	0.7868	0.7272	0.8134	0.7868	0.7272	0.8134	0.7868	0.7272
**CosPlus**	0.6975	0.7409	0.7192	0.6975	0.7409	0.7192	0.6975	0.7409	0.7192	0.6975	0.7409	0.7192	0.6975	0.7409	0.7192
**RWR .85**	0.5988	0.7321	0.5	0.5988	0.7321	0.5	0.5988	0.7321	0.5	0.5988	0.7321	0.5	0.5988	0.7321	0.5
**∼.95**	0.5994	0.7331	0.5	0.5994	0.7331	0.5	0.5994	0.7331	0.5	0.5994	0.7331	0.5	0.5994	0.7331	0.5
**SimR**	0.7019	0.8202	0.5	0.7019	0.8202	0.5	0.7019	0.8202	0.5	0.7019	0.8202	0.5	0.7019	0.8202	0.5
**LRW _3**	0.6607	0.8054	0.4462	0.6607	0.8054	0.4462	0.6607	0.8054	0.4462	0.6607	0.8054	0.4462	0.6607	0.8054	0.4462
**∼_4**	0.7033	0.8255	0.4462	0.7033	0.8255	0.4462	0.7033	0.8255	0.4462	0.7033	0.8255	0.4462	0.7033	0.8255	0.4462
**∼_5**	0.7376	**0.8443**	0.7335	0.7376	**0.8443**	0.7335	0.7376	0.8443	0.7335	0.7376	0.8443	0.7335	0.7376	0.8443	0.7335
**SRW_3**	0.6606	0.8042	0.4462	0.6606	0.8042	0.4462	0.6606	0.8042	0.4462	0.6606	0.8042	0.4462	0.6606	0.8042	0.4462
**∼_4**	0.7034	0.8261	0.4462	0.7034	0.8261	0.4462	0.7034	0.8261	0.4462	0.7034	0.8261	0.4462	0.7034	0.8261	0.4462
**∼_5**	0.7376	0.8432	0.7334	0.7376	0.8432	0.7334	0.7376	0.8432	0.7334	0.7376	0.8432	0.7334	0.7376	0.8432	0.7334
**MFI**	0.5978	0.7302	0.5	0.5978	0.7302	0.5	0.5978	0.7302	0.5	0.5978	0.7302	0.5	0.5978	0.7302	0.5
**TSCN**	0.5906	0.7196	0.5	0.5906	0.7196	0.5	0.5906	0.7196	0.5	0.5906	0.7196	0.5	0.5906	0.7196	0.5
**Prone**	0.7389	0.6995	0.647	0.7864	0.6803	0.7269	0.8373	0.8129	0.7158	0.9486	0.9346	0.9063	**0.9405**	**0.9108**	**0.8854**
**NetMF**	0.7548	0.7262	0.692	0.4916	0.5078	0.5156	**0.8879**	**0.8826**	**0.8182**	**0.955**	**0.941**	**0.9238**	0.854	0.8435	0.8025
**Node2vec**	0.5898	0.5621	0.5153	0.5819	0.5175	0.5646	0.6973	0.6457	0.5543	0.9157	0.8864	0.8526	0.8065	0.7876	0.7357
**Deepwalk**	0.5871	0.5592	0.5098	0.5805	0.516	0.5657	0.698	0.6463	0.5548	0.9151	0.8863	0.8491	0.8057	0.7869	0.7336
**Hope**	0.6304	0.5313	0.5542	0.6086	0.5542	0.6075	0.7318	0.6172	0.6128	0.8304	0.7457	0.7841	0.8894	0.866	0.8076
**NetSMF**	0.5721	0.5282	0.5153	0.3822	0.3994	0.4111	0.7191	0.6449	0.5987	0.7923	0.7191	0.7431	0.7095	0.6736	0.6733
**Grarep**	0.0724	0.277	0	0.2765	0.3671	0.318	0.3796	0.3252	0.1624	0.2992	0.3522	0.2538	0.6739	0.6286	0.6435
